# Relevant factors affecting nurse staffing: a qualitative study from the perspective of nursing managers

**DOI:** 10.3389/fpubh.2024.1448871

**Published:** 2024-08-14

**Authors:** Gege Li, Waner Wang, Jiangfeng Pu, Zhanghao Xie, Yixuan Xu, Tiemei Shen, Huigen Huang

**Affiliations:** ^1^Department of Nursing, Jinan University, Guangzhou, China; ^2^Department of Nursing, Guangdong Provincial People’s Hospital (Guangdong Academy of Medical Sciences), Southern Medical University, Guangzhou, China; ^3^Department of Nursing, Guangdong Pharmaceutical University, Guangzhou, China; ^4^Department of Nursing, Shantou University Medical College, Shantou, China

**Keywords:** nurse staffing, nurse manager, semi-structured interviews, influencing factor, human resources

## Abstract

**Objective:**

To understand the current situation of nursing manpower allocation, explore the factors affecting nurse staffing, improve nurse staffing level, and provide reference for better formulation of nursing human resources staffing standards.

**Methods:**

A descriptive research method was used to conduct semi-structured interviews with 14 nursing managers. The data were analyzed and refined by content analysis. The sample size was subject to content saturation.

**Results:**

Nine themes and twenty sub-themes of influencing factors for nursing staffing were identified across four levels: hospital level, department level, patient level, and nurse level.

**Conclusion:**

Hospital and department managers need to comprehensively consider the factors of affecting nurse staffing. Adopting multidimensional optimization measures, improving relevant systems, optimizing nurse structure, and establishing flexible and mobile nurse database to cope with public emergencies, so as to effectively improve nurse staffing and nursing service quality.

## 1 Introduction

The global nursing workforce shortage has been a major challenge in the medical field. In 2020, the World Health Organization reported a shortage of 5.7 million nurses and midwives (1). By the end of 2022, the total number of health workers in China was 14.411 million. Among them, the total number of registered nurses is 5.224 million (2). Compared with the 2025 China nursing career planning target, there is still a gap of 276,000 (3). The number of registered nurses in China in 2022 is 3.17 per 1,000 population, which is far lower than the global median density proposed by WHO (4.86 per 1,000 population) (4). In the evolving healthcare sector, ensuring optimal nurse staffing remains a key issue in hospital administration and nursing management (5, 6). Adequate nurse staffing is critical not only to provide high quality patient care, but also to maintain nurse job satisfaction and reduce turnover (7, 8). However, the factors that influence nurse staffing are multifaceted and complex, including a range of variables from institutional policies to the characteristics of individual nurses. At present, researchers mainly focus on quantitative studies on nurse staffing and nursing quality, patient outcomes and nurse outcomes (9, 10). However, there are few qualitative studies on the influencing factors of nurse staffing. Nursing managers play a key role in staffing decisions, pay close attention to day-to-day operational dynamics and challenges, and are uniquely positioned to provide insights into the various factors that affect nurse staffing (11). Therefore, this study aims to explore the influencing factors of nurse staffing through the perspective of nursing managers, and through in-depth interviews and thematic analysis, reveal the human resource challenges and considerations faced by nursing managers in their efforts to ensure appropriate nurse staffing levels. The results of this study will help develop more effective staffing strategies and policies, ultimately improving the quality of care provided to patients and improving the working environment for nurses.

## 2 Research objectives

This qualitative study explores the influencing factors of nurse staffing. Two nursing managers were selected for pre-interview, and the final interview outline was revised according to the feedback of the pre-interview. See Table 1 for details.

**Table 1:** Interview outline.

Question
1 What is the current situation of nursing staffing in your ward? And what do you think of the current situation of nurse staffing?
2 What problems do you encounter when staffing? And how did you solve these problems?
3 What do you think are the factors that affect the staffing of nurses?
4 What are your suggestions for nursing staffing?

## 3 Materials and methods

### 3.1 Participant recruitment

From December 2023 to February 2024, nursing managers in a Grade 3 hospital in Guangdong Province were selected as interview subjects by purpose sampling method. Inclusion criteria: (a) Engaged in clinical work ≥10 years; (b) Engaged in management work ≥3 years; (c) have intermediate or above professional titles; (d) Informed consent and voluntary participation in this study.

### 3.2 Data collection methods

The development of the interview outline was based on the research objectives and a review of relevant literature, following a systematic process (12). An initial draft of the interview guide was formulated to focus on the research theme. This draft was refined through discussions in project team meetings. Before the formal interviews, two nursing managers were invited to participate in pilot interviews, and since no further revisions were needed, the pilot interview data were included in the subsequent analysis. The qualitative research involved conducting semi-structured, in-depth face-to-face interviews with the participants. Before each interview, one researcher (LGG) obtained informed consent from the interviewees, explaining the purpose, methods, and content of the interview. The interviews were recorded and noted, and were conducted in the interviewee’s office to ensure privacy. To protect the confidentiality of the participants, two researchers (LGG and WWE) were responsible for questioning and recording, respectively, using pseudonyms N1 to N14 instead of real names. The interviews were conducted in easily understandable language, encouraging participants to express their feelings and thoughts fully. Active listening, clarification, and probing techniques were employed, along with noting non-verbal cues. Each interview lasted between 30 and 60 min, with an average duration of 39 min.

### 3.3 Data analysis methods

Data collection and analysis in this study were conducted simultaneously. Within 48 h after each interview, the recordings were transcribed verbatim, and the non-verbal information recorded during the interviews was integrated into the documents, forming complete interview transcripts. We used thematic analysis to analyze the data (13), organizing the materials with NVivo Plus 11 software. Throughout the analysis process, the two researchers (LGG and WWE) maintained an open and neutral attitude, repeatedly reading the transcripts and non-verbal notes. By continuously comparing and inductively analyzing the data, we extracted themes to gain a deeper understanding of the participants’ actual meanings. Text from individual interviews was divided into meaning units that were condensed and coded. The themes in the table were derived from the initial codes, with similar codes grouped into subcategories and categories, which were then further organized into themes (14). The first author identified the initial codes, while the other authors reviewed the coded interview samples. Any disagreements regarding themes were resolved through discussions among all authors until a consensus was reached.

## 4 Results

### 4.1 Sample size

The sample size was by information saturation (15). Finally, a total of 14 participants participated in this study, among which 13 were female and 1 was male, and the average age of all participants was 45.86 years old (33 ~ 52 years old). The average duration of clinical work was 25.57 years (11–23 years) and the average duration of managerial work was 11.21 years (4–16 years). The demographic characteristics of participants are shown in Table 2.

**Table 2:** General data sheet for participants.

ID	Gender	Age	Educational level	Professional title	Clinical working time	Manage working time
N1	Female	33	Bachelor	Supervisor nurse	11	6
N2	Female	52	Bachelor	Supervisor nurse	34	16
N3	Female	46	Bachelor	Chief nurse	21	12
N4	Female	45	Bachelor	Deputy chief nurse	21	10
N5	Female	48	Bachelor	Deputy chief nurse	21	10
N6	Female	44	Bachelor	Supervisor nurse	21	9
N7	Female	50	Bachelor	Deputy chief nurse	22	15
N8	Female	47	Bachelor	Deputy chief nurse	21	12
N9	Female	51	Bachelor	Deputy chief nurse	23	15
N10	Female	50	Bachelor	Supervisor nurse	23	14
N11	Female	42	Bachelor	Deputy chief nurse	18	4
N12	Male	39	Bachelor	Deputy chief nurse	16	9
N13	Female	47	Bachelor	Supervisor nurse	21	12
N14	Female	48	Bachelor	Deputy chief nurse	22	12

### 4.2 Hospital level

#### 4.2.1 Imperfect policies and systems

Decisions made by hospital management based on national policies, such as establishing standards for bed-to-nurse ratios in each department, inspection systems, training and promotion mechanisms, significantly impact nurse allocation. However, N12 highlights that for specialized departments like emergency care, there lacks a unified allocation standard in China (16), which complicates nurse allocation levels to some extent. Several respondents in the study noted limitations in current nurse staffing due to rigid adherence to bed-to-nurse ratios that often fall short, thereby compromising adequate staffing levels. Furthermore, the imperfect inspection, promotion, and training systems in the face of a complex and evolving clinical environment constrain nurses’ workload and career advancement opportunities, affecting both stability and quality of nursing care.

#### 4.2.2 Insufficient manpower reserve

The hospital’s human resource reserve directly influences the quantity and quality of nurses, as well as the hospital’s capacity to address staffing shortages, temporary needs, and emergencies. Respondents in the study also noted that due to insufficient or lacking hospital manpower reserves, even submitted personnel demand applications often remain unresolved.

### 4.3 Department level

#### 4.3.1 Department characteristics

In the rational allocation of nursing human resources, it is necessary to consider a variety of factors, such as the department’s patient conditions, department size, bed turnover, workload, and the need for specialized nursing. For instance, departments like emergency, intensive care, and surgery require nurses with specific professional skills and knowledge to ensure patient safety and effective treatment outcomes.

#### 4.3.2 Cost-effectiveness driven nurse staffing

Reasonable manpower cost control can be in to ensure the quality of care under the premise of optimizing the allocation of resources, improve the efficiency of the cost. Appropriate labor cost control of department, fair and reasonable scheduling system, by ensuring reasonable salary, meet the demand of nurses reasonable help stabilize the nurse team, reduce turnover and maintain the continuity and stability of the nursing work, at the same time maximize the cost-effectiveness of department, ensure quality of nursing service.

#### 4.3.3 Cultural construction of the department

Department culture construction is one of the important factors affecting the allocation of nurses, including good working atmosphere and teamwork, effective communication and mutual understanding, and staff care. A good department culture construction can improve the satisfaction and stability of nurses, and ensure the efficiency and high quality of nursing work. In the study, respondent N1 also said that a good working atmosphere in the department would alleviate the original shortage of manpower.

#### 4.3.4 Multi-level recognition and support

Nurses occupy a large proportion in the department and play an indispensable role. Recognition at the department level, including but not limited to the head nurse and department director, as well as the recognition and support from doctors for the work of nurses, can not only enhance nurses’ job satisfaction and loyalty but also significantly improve the efficiency and quality of nursing care. This influence helps optimize working conditions for nurses.

### 4.4 Patient level

#### 4.4.1 Patient profile

With the rapid development of social economy, the demand for nursing services is gradually increasing. The basic situation of patients, including the number of patients, the complexity of the disease, the self-care ability of patients and the patient’s needs, which will virtually affect the workload of nurses, and therefore put forward requirements for the staffing of nurses.

### 4.5 Nurse level

#### 4.5.1 Challenges in nursing human resources

The shortage of nurses is a significant issue in nursing human resource management, profoundly affecting nurse staffing. Insufficient nursing staff can lead to a decline in the quality of nursing services, failing to meet patient care needs, and thereby impacting treatment outcomes and patient satisfaction. Respondents to the study indicated that this shortage is expected to persist in the short term. Currently, nurses face high workloads and pressure, with long-term overwork increasing their physical and psychological burden, reducing work efficiency and quality, and leading to higher turnover rates. This exacerbates the challenges in nurse staffing.

#### 4.5.2 Nursing professional development

The structure of nurses is an important part of human resource management, including the age, work experience, title, comprehensive ability and specialized skills of nurses. Optimizing the structure of nurses can promote the staffing of nurses. Provide continuous professional training and career development opportunities for the nursing team, including advanced studies, promotions, and specialized training. These initiatives will improve nurses’ professional skills and career satisfaction, enhancing their willingness to stay in the department and their overall stability (see Table 3).

**Table 3:** Influencing factors of nursing staffing.

First-level themes	Second-level themes	Nursing manager quote
Hospital level		
Imperfect policies and systems	Lack of unified standards for emergency nurses	N12:"There’s no national standard for emergency staffing, and we have talked about it, but there’s no clear target for staffing, so nurse staffing levels are definitely going to be affected.”
	Limitations of bed-to-nurse ratio standards	N2:"the current department is not up to the standard of the hospital’s bed-to-care ratio… Even with the deployment of mobile nurses and nurses from other departments, they are still unable to fill the gap.”
		N9:"Our department is the largest department in the hospital, according to the designated bed-to-nurse ratio, we need to reach 39 nurses, but currently there are only 35, and when we add the nurses who resign, retire or take maternity leave, the department’s manpower is very tight.”
		N13:"Relatively speaking, our department did not meet the hospital’s standard for bed-to-nurse ratio. Our department is unique in that nurses are also assigned to the respiratory center and outpatient department, resulting in fewer nurses available for the wards and tighter manpower resources.”
		N14:"Not every department in our hospital has established a standard for nurse-to-bed ratio, but currently, we are unable to meet this ratio and face insufficient manpower.”
	Frequent checks, and imperfect promotion and training systems	N3:"Because A Class III Grade A hospital, often face too many examinations, including our own nursing department, the hospital’s, and the state’s examinations, minimizing the pressure of such examinations on nurses, and really give the nurses back to the patient, I think the nurse is actually very willing.”
		N9:” In the area of management, I think it is possible to relax the training conditions for this specialist nurse?… Such a situation exists in our department, the ability can be reached, but the academic degree cannot be reached…”
Insufficient manpower reserve	Inability to cope with staff shortages in emergency cases	N5:"During the COVID-19 epidemic, many nurses have gone out, they can only ensure safety, and the rest cannot be managed at all… In the case of extreme manpower shortage, only basic work can be ensured “
		N8:"But now, when a member of the department is sick, or there are emergencies, or even nurses need to be placed on leave, in such cases, other nurses have to work overtime because there is no other staff to supplement.”
	Inability to timely address manpower shortages	N6:"I have applied to the head nurse of the department, but the head nurse answered that many departments are short of staff, which may be difficult to solve, so I did not apply to the above, indicating that this problem may indeed exist in the past two years.”
		N2: “There is no way, you can only wait for a new group of nurses, or in fact, the hospital has mobile nurses, but because of the two-way selection policy, many nurses will not choose the workload of the department, it is still difficult.”
Department level		
Department characteristics	Specialty nursing needs and technology	N1: “Our specialty has a higher risk of mental violence, which affects nurse staffing. Considering the risk factors, it is best to have a mix of male and female nurses. However, there are very few male nurses, resulting in a greater demand for them.”
		N11: “The current staffing is based on existing manpower, but the ICU is basically a very serious patient, and the condition can change at any time… Therefore, manpower will be taken into account “
		N12:"Because our emergency department operates under fixed conditions, our staffing requirements differ significantly from other departments. I need to consider many additional factors.”
	Departmental work	N5: “This time will be better, because it is near the New Year, and our department basically receive more elective surgeries… So it obviously seems that during this period, the patient beds are a little empty, so the manpower is not so tight “
		N9: “It will also take into account the working hours, and allocate personnel to work according to the situation of the time period… And gynecological preoperative preparation is very time-consuming, and each department has different specialty characteristics.”
Cost-effectiveness driven nurse staffing	Control of nurse labor costs	N1:"In accordance with the hospital provisions of the bed protection ratio standard, the department is currently able to operate, because there are too many manpower, the department’s income is not high, then everyone’s income will be lower… So the current manpower will have little impact on people’s income.”
		N3:"As nursing manager, I also do not want too many of my nurses… It is good to be able to meet the rotation of departments.”
		N11: “Due to better departmental economic performance, the department bonuses are relatively high, contributing to higher job satisfaction among the nurses.”
	Equitable and rational scheduling system	N1: “In terms of scheduling, fairness is a priority in our department due to varying workloads and flexible assignments, aiming to achieve a balanced approach.”
		N2: “Our department has maintained a rotating leave system for a long time, emphasizing the importance of fairness and considering each nurse’s needs when scheduling.”
		N6: “When it comes to scheduling, we prioritize balance and fairness. It’s not about one nurse’s workload preventing others from taking leave; rather, we strive to ensure equitable opportunities for all.”
		N13: “In our department, vacation scheduling adheres strictly to established rules. Everyone is conscientious about following these rules, ensuring a humanized approach to scheduling.”
Cultural construction of the department	Positive department atmosphere and team collaboration	N1:"The atmosphere of our department is very good. If a department has a strong cohesion, it is easier to overcome and solve any problems. Even in the absence of foreign assistance, we can also overcome the shortage of manpower.”
		N11: “In the department, everyone helps each other, so that things can be dealt with quickly and the work efficiency is high. If you share a thing with everyone, the work will be much easier, so the working atmosphere is still very important.”
		N13:"In fact, our nurses are not well treated at the moment, so team building is very important. First, as managers, we must ensure the physical and mental health of these nurses, but also put humanistic care for nurses first.”
	Effective communication and mutual understanding	N1: “Colleagues need to truly understand each other. The higher the cohesion in a department, the more likely they are to spontaneously solve many problems.”
		N6: “If a nurse in our department faces a significant issue at home, everyone works hard to help resolve it. I think this is a wonderful aspect of our team.”
		N14: “Our nurses are quite aware. If one nurse is very busy while others are unresponsive, I, as a manager, will communicate and ask everyone to help. After doing this a few times, everyone naturally starts to help out voluntarily. So, timely communication is essential.”
	Humanistic care in nursing	N5: “The atmosphere of our entire department I think is good, because if there is any problem, everyone will solve it together, I’m not gonna do this alone … I think the whole department has a good feeling, and the young people are also more motivated.”
		N9: “To foster a positive working atmosphere, as managers, we should pay close attention to nurses’ emotions, communicate promptly, and provide appropriate comfort when needed.”
		N10: “We will hold some team building activities regularly, and we will care about the family status of nurses… So the atmosphere in the department is very good. It is also important for the team to draw on the strengths of the nurses and provide timely encouragement and support.”
Multi-level recognition and support	Leadership of recognition and support	N3: “The daily work of clinical nurses is very hard, and we hope to increase the value of nurses… In fact, as managers, we are more respectful of the nurses’ willing, and the head nurse is actually just an executor. We should learn to think from the perspective of nurses, so that nurses can reduce the mentality of boredom or resignation.”
		N8: “When tasks are assigned to nurses within the department, it is important to acknowledge and appreciate their work. This recognition makes the nurses feel valued and supported.”
		N14: “I believe recognizing nurses’ work is crucial. It helps to utilize each nurse’s strengths, fostering a sense of responsibility and allowing them to realize their self-worth.”
	Doctors’ understanding and support	N3: “Doctors recognize and support the hard work and contributions of nurses. Nurses greatly need this validation and partnership, as it fosters a more enjoyable and positive work atmosphere.”
		N6: “Then the director of our department is still very good and very supportive of our nursing.”
Patient level		
Patients profiles	Patient volume and condition	N2: “The patient’s condition must be taken into account, and another aspect is the patient’s self-care ability.”
		N3:"Depending on the number of patients, and also depending on the patient’s condition, if the illness is very serious, maybe you can not just the original number of shifts, sometimes necessary, really have to start some programs to help…”
		N6:"In the past, our department primarily focused on hepatobiliary surgery, where patients generally had better prognoses and basic conditions. Now, the department has shifted to pancreatic surgery, which is the most complex and severe operation in general surgery, with patients often having the most complications and poorer overall conditions.”
		N10: “According to the needs of the patients, the number of patients and the severity of the disease.”
	The patient’s needs	N4: “The first thing I will consider is the patient. I want to take good care of them by prioritizing their needs, which will guide our personnel.”
		N8: “We must certainly prioritize the patient’s needs. The patient’s needs and safety come first, while also considering our existing manpower and the demands on our nurses.”
		N9: “Of course, the staff should prioritize the patient, using the patient’s needs as a guide.”
		N13: “I believe the current schedule is more suitable for our families, especially in pediatrics. Unlike other departments, pediatrics requires a significant amount of work. Parental expectations are relatively high, and meeting these expectations is crucial.”
		N14: “Staffing is available, but we must also consider patient needs. It’s important to keep patients safe.”
Nurse level		
Challenges in nursing human resources	Shortage of nurses	N2: “The shortage of nurses is also a problem facing the whole hospital. It’s not that I cannot make up for you, but I just cannot make up for so many people to give you. It turned out that there were not so many people, and then it was distributed, but it was still not enough.”
		N3: “Manpower is insufficient, I can only compress shifts… And the workload… That workload is transferred to the responsible nurses.”
		N8: “In terms of quantity, the hospital’s staffing standards are still not met, and the number of nurses is still not enough…”
		N10:"According to hospital standards, our department currently lacks two nurses, and one nurse is on maternity leave. If the two nurses who are nearing retirement also leave, the department will face challenges in scheduling shifts. Even if a new nurse is assigned to the department, she will require at least three months of training before being able to work independently.”
	Nurses are overloaded	N7: “In terms of quantity, the hospital’s staffing standards are still not met, and the number of nurses is still not enough…”
		N9: “Nurses report less rest, they cannot guarantee two days off a week, the second is busy work, long working hours, often delayed work… Then they will definitely complain, plus our low income, it will be even more dissatisfied, and there may be a problem in terms of turnover.”
		N11: “Then there are some other, temporary specialist assignments in our department… Because I do not have a specialist nurse, I do not have a full-time nurse at the moment, so I have to deploy in this group… So this is a problem in our daily work, and sometimes it is a difficult management problem.”
Nursing professional development	Skill set of nurses	N2:"Then there are some other, temporary specialist assignments in our department… Because I do not have a specialist nurse, I do not have a full-time nurse at the moment, so I have to deploy in this group… So this is a problem in our daily work, and sometimes it is a difficult management problem.”
		N4: “Years of work are the main thing, and the ability to work…”
		N5:"The first to consider the nurse’s communication … And then, when appropriate, you have to look at the mix of people.”
		N8: “The communication ability of nurses also depends on the personal ability and character of nurses.”
	Personal career development	N7: “There are also some successors of head nurses and specialist nurses who should be encouraged to continue to upgrade their academic qualifications… The head nurse should also train these talents, otherwise, the department will not be able to find a successor when the head nurse retires, and we must train talents.”
		N11: “I am encouraged by the opportunity for new studies and equal chances to explore, as this was my own experience. As long as you are eager to advance, I will provide more opportunities. It’s important for young people to learn and broaden their perspectives on the world.”
		N13: “I think the whole word… Encourage them more, take more classes, let them know that career prospects are better, reduce turnover.”

## 5 Discussion

For additional requirements for specific article types and further information please refer to “Article types” on every Frontiers journal page. From the perspective of nursing managers, this study discussed the relevant factors affecting nurse staffing, mainly from four levels: hospital, department, patient and nurse level.

The results of this study indicate that nurse staffing is related to the imperfection of hospital policies and systems, the lack or insufficiency of manpower reserves, and the control of nurse manpower costs. The formulation of policies related to nursing human resources can play a crucial role in the number of nurses. On the one hand, although China has formulated many standards, principles and plans for staffing (17–20), but at present, most medical institutions in our country still adopt a single number of beds for human staffing, ignoring the differences of condition, disease, service quantity, etc., which makes it difficult to meet the clinical practical needs of nursing human resources. On the other hand, the participants indicated that the hospital did not reserve talents or had insufficient reserves at present, and the study found that public emergencies would have a certain impact on the staffing of nurses, and the reasonable establishment of a mobile nurse base could make up for the shortage of manpower to a large extent in crisis situations (21).

At present, there is no legislation on nurse staffing in China, but there are a few areas (10, 22, 23), such as Victoria or Queensland in Australia and California in the United States, have made the nurse–patient ratio mandatory, and scientifically found that this measure is beneficial to patients and healthcare systems. Belgium is also reforming its nursing staffing policy and using part of its budget to hire non-nursing staff to alleviate the shortage of nurses, while India is making further efforts to revise the standards of the nurse-to-patient ratio. Yet labor costs dominate hospital budgets. They are easy targets for cuts to offset other expenses (24). The financial budget of a hospital on the cost of nursing manpower will directly affect the number of nurses, resulting in the imbalance of the nurse-to-patient ratio, and increasing the work pressure and load of existing nurses (25). In the future, when developing safe and reasonable nurse staffing, it is necessary to comprehensively consider the control of nurse labor cost and solve the problem of baseline nurse staffing, so as to better cope with the fluctuation of nurse nursing demand among patients (26, 27).

In addition, the shortage of manpower reserve makes it difficult for hospitals to quickly deploy enough nurses in the face of emergencies (such as epidemics, natural disasters, etc.), which affects the timeliness and effectiveness of nursing work. First of all, in order to actively and effectively respond to public emergencies and other emergency events, it is necessary to establish a mobile nurse team. At the same time, it is necessary to strengthen the hospital’s leadership of the nurse team and improve the emergency level of the nurse team. Secondly, the recruitment process of new nurses should be accelerated, and the relevant training mechanism should be optimized to ensure that new nurses can quickly get on the job and adapt to the work needs. Finally, establish reasonable vacation and prepare class arrangements, to ensure that the nurse has enough during the period of vacation or sick leave substitute nurses to fill the gap, to ensure the safety of patients and the high quality nursing service.

The results of this study show that the staffing of nurses is related to the department characteristics, cost-effectiveness driven nurse staffing, department culture construction and multi-level recognition and support. Nurses with affected by the characteristics of the various specialist departments for college work, care needs and technical differences, nurse need to change accordingly, in order to ensure safe and effective nursing service. The control of nurse labor costs varies between departments and hospitals due to differing economic benefits. It is crucial to balance the relationship between existing nurse staffing and costs. A limited department budget can affect the hiring of additional nurses or the ability to increase nurses’ salaries, directly impacting nurse staffing levels. Implementing a fair and reasonable scheduling system ensures that each nurse receives adequate work and rest time, preventing overwork or underwork. This can improve job satisfaction and work efficiency, thereby reducing nurse turnover rates and fatigue (28).

Additionally, fostering a positive departmental culture is also essential. Nurses’ working environment and atmosphere, and leadership style can affect nurses’ turnover intention, thus further affecting the staffing level of nurses and the quality of nursing work by affecting nurses’ turnover intention (29–31). Active teamwork, effective communication, and mutual understanding are crucial factors in optimizing nurse allocation and improving the quality of nursing services. Good teamwork and effective communication streamline workflow, reduce redundant tasks and communication errors, and enhance nursing efficiency (32, 33). Mutual understanding and trust among team members facilitate knowledge and experience sharing, promoting both individual and team skill development. A well-functioning department resembles a warm, supportive family, which requires managers to show concern and care for nurses, focusing on their physical and mental health needs. This helps alleviate work pressure, strengthens colleague relationships, and enhances team stability. Multi-level recognition and support from department directors, head nurses, doctors, and patients serve as key motivators for nurses, reinforcing their commitment and sense of value in their work. However, the lack of necessary material and emotional support increases job burnout and affects nurses’ intention to stay (34, 35). Therefore, in this challenging environment, it is crucial to optimize nurse allocation, enhance the quality of care, and ensure the stability of the nursing team. This can be achieved by improving the working environment and atmosphere, fostering team cooperation, formulating reasonable scheduling plans, and establishing effective communication and feedback mechanisms.

With the accelerated aging of China’s population and the increasing prevalence of chronic diseases, the demand for long-term care and health management has risen significantly. The growing number of patients directly impacts the number of nursing staff needed in departments. A high patient load necessitates more nursing staff to ensure each patient receives adequate care. This situation poses a challenge for managers in nursing staff allocation, requiring flexible adjustments based on patient admissions and discharges to respond to fluctuating peaks and troughs.

Patients with complex conditions require higher levels of nursing skills and more hours of care, necessitating additional specialized nursing staff or more training and support. As patients’ expectations for the quality of nursing care rise, departments need to increase their nursing staff to meet these expectations. A higher level of nursing expertise can improve patient satisfaction, delivering higher quality care and greater value (36). Therefore, when planning nurse allocation, managers should consider the number of patients, the complexity of conditions, and specific nursing needs. By conducting thorough evaluations and making flexible adjustments to the nursing team, managers can effectively address the needs of different patient groups and enhance the quality and efficiency of nursing services.

Nurses are the largest group in the medical and health system, occupying an irreplaceable position, and sufficient nursing staff is the premise and basis of rational allocation of nursing human resources. The shortage of nurses is a serious problem facing the world today (37, 38). The shortage of nurses will lead to the increase of nursing workload and labor intensity, resulting in a high turnover rate of nurses, and turnover intention is a predictive factor of turnover rate (39, 40). The turnover intention of nurses varies significantly among different countries. The turnover intention of nurses in South Korea was 18.8% (41), 22.5% of nurses in European countries expressed their intention to quit (42) and 43% of nurses in Lebanon expressed an intention to leave within one year (43). In China, the turnover intention of nurses in East China is 43% (44) and that of nurses in Guangdong Province is as high as 64.1% (40), which means that the turnover rate of nurses in China remains high, and the gap of nurses will further increase. Attracting and retaining the existing nurse workforce is critical to maintaining high quality patient care (26). Studies have shown that nurses are the foundation of patient safety and nursing quality, and higher nurses’ satisfaction will bring better job performance, nursing quality and employee retention (34, 45). In addition, research shows that with adequate nurse staffing, good working environment and welfare benefits, nurses will have higher job satisfaction and lower turnover intention, thus ensuring the stability of the nurse team and nursing quality (46). With the continuous improvement of medical technology, higher requirements are put forward for the education level and working ability of nurses. Nurses need to constantly enrich their theoretical knowledge and improve their nursing skills in order to meet the growing nursing needs and the speed of high-quality development of hospitals. Therefore, the nurse structure should be considered and further optimized when staffing nurses.

### 5.1 Limitations

The study has several limitations. First of all, in order to ensure the diversity of the study subjects, we used purpose sampling to sample the head nurses of various departments in the hospital, but the study scope was limited to one hospital, which made it difficult to obtain additional information. Secondly, the interview document data is translated from Chinese to English, and there is a certain risk of translation errors.

## 6 Conclusion

From the perspective of nursing managers, this study explores the related factors affecting the allocation of nurses through descriptive qualitative research, explores the specific challenges, pressures and needs of nurses in their work, and reveals the deep causes of the shortage of nurses from multiple levels and directions, rather than just stay on the quantity and statistical data. From a nursing manager’s perspective, the results of this qualitative study highlight the multifaceted and interrelated factors that influence nurse staffing. Hospital-level factors are the most critical. Improving and unifying nursing personnel staffing standards is the premise of ensuring the development of high-quality nursing. Nursing managers stressed the importance of a supportive work environment, effective communication, and continuing professional development to mitigate staffing challenges. At the same time, it reveals the influence of humanistic and emotional factors, and captures the importance of humanistic care, emotional support and professional identity experienced by nurses in the work. These factors have an important impact on the job satisfaction and retention rate of nurses, thereby indirectly affecting the staffing of nurses. Addressing these factors through integrated strategies can improve nurse retention, improve the quality of patient care, and foster a more stable and satisfied nursing workforce. This study can provide deep insight and effective strategy suggestions for understanding and solving today’s nurse staffing problems, so as to provide a unique contribution to the continuous development and improvement of the nursing profession.

## Data availability statement

The original contributions presented in the study are included in the article/supplementary material, further inquiries can be directed to the corresponding author.

## Ethics statement

The studies involving humans were approved by Ethics Review Committee of Guangdong Provincial People’s Hospital. The studies were conducted in accordance with the local legislation and institutional requirements. The participants provided their written informed consent to participate in this study. Written informed consent was obtained from the individual(s) for the publication of any potentially identifiable images or data included in this article.

## Author contributions

GL: Conceptualization, Data curation, Formal analysis, Investigation, Visualization, Writing – original draft, Writing – review & editing. WW: Conceptualization, Data curation, Formal analysis, Investigation, Writing – review & editing. JP: Conceptualization, Data curation, Writing – review & editing. ZX: Supervision, Writing – review & editing. YX: Methodology, Supervision, Writing – review & editing. TS: Methodology, Project administration, Supervision, Writing – review & editing. HH: Supervision, Writing – review & editing, Funding acquisition, Resources.
